# Supplementary figures and images for: Facilitating behavioral change: A comparative assessment of ASHA efficacy in rural Bihar
Source: PLOS Glob Public Health. 2022 Aug 17;2(8):e0000756. doi: 10.1371/journal.pgph.0000756 (PMC10021476; doi:10.1371/journal.pgph.0000756)

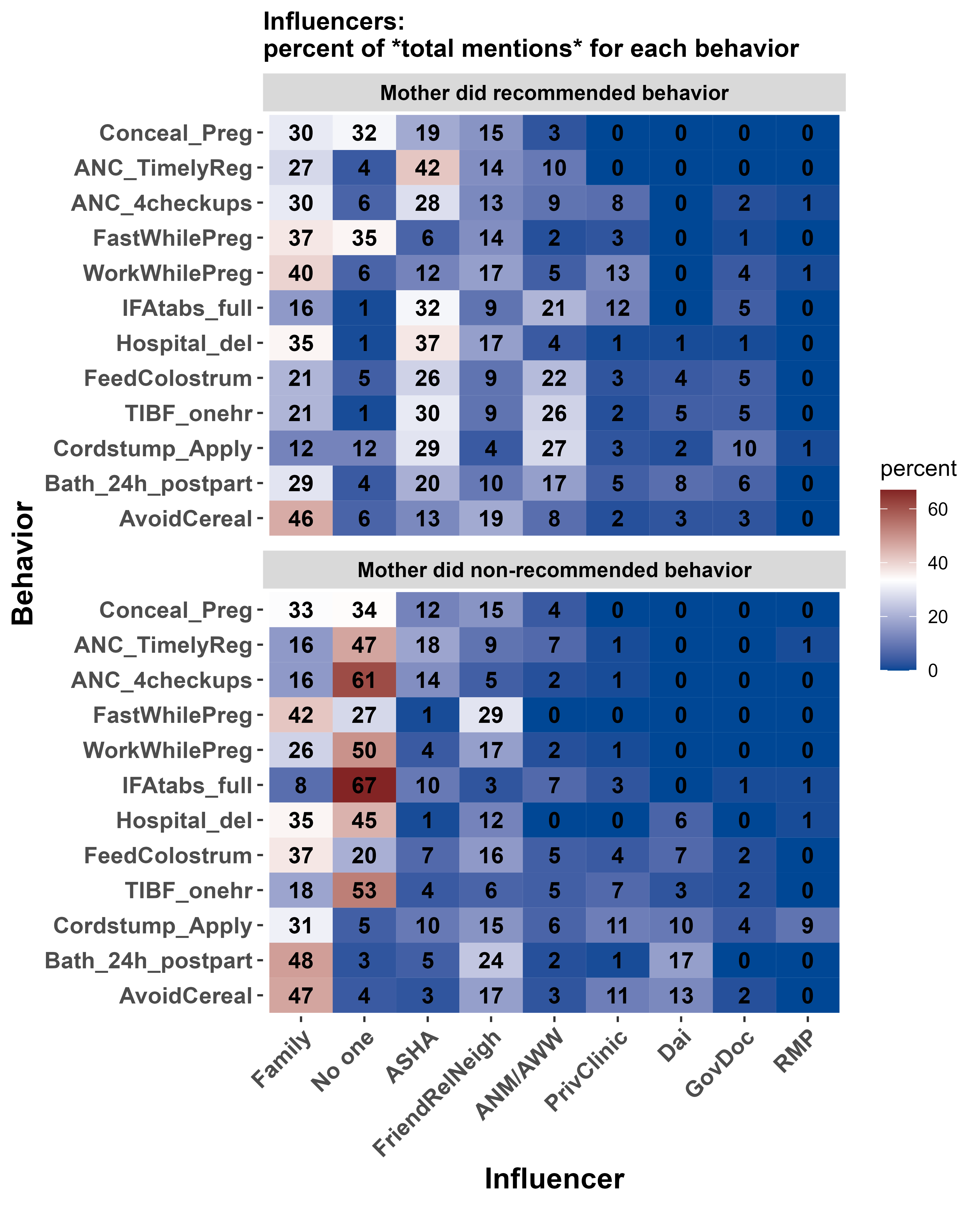

Supplement: S1 Fig — Behaviors are listed roughly sequentially from top to bottom on the y-axis. Influencers are ordered by the overall frequency of mention from left to right on the y-axis such that more frequently mentioned influencers are to the left. (TIF) [file pgph.0000756.s001.tif]

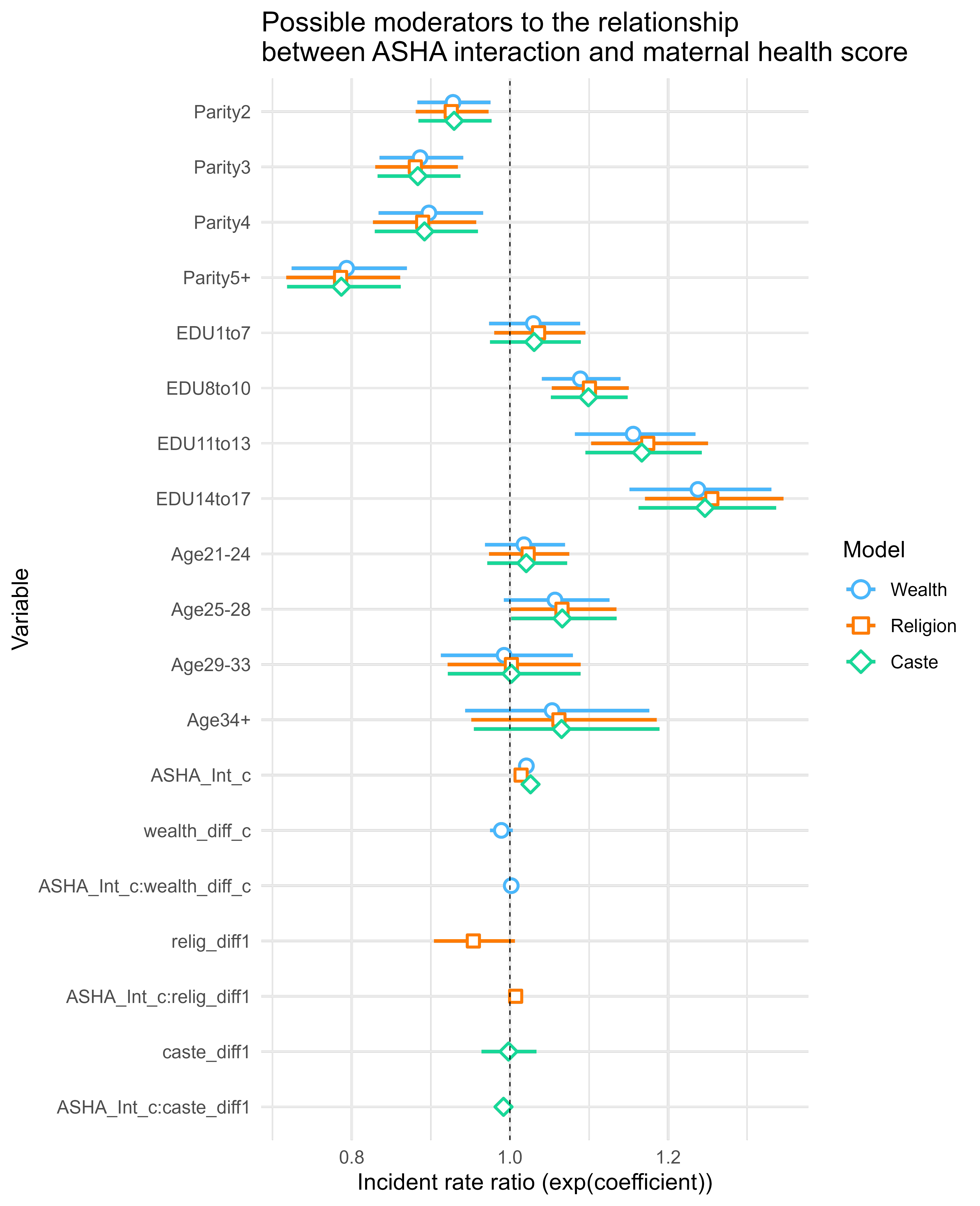

Supplement: S2 Fig — Coefficients for each control variable, potential moderator (wealth, caste, and religion), ASHA interaction score, and interactions between moderators and ASHA interaction score. (TIF) [file pgph.0000756.s002.tif]
