# Supplementary material for: Facilitating behavioral change: A comparative assessment of ASHA efficacy in rural Bihar
Source: PLOS Glob Public Health. 2022 Aug 17;2(8):e0000756. doi: 10.1371/journal.pgph.0000756 (PMC10021476; doi:10.1371/journal.pgph.0000756)
Supplement: S1 Table — (DOCX) [file pgph.0000756.s003.docx]

Table S1: Counts for yes/no questions on the ASHA interaction score

| Question | count |
| --- | --- |
| Q220 When you were pregnant, did asha come to your home to meet you anytime during the pregnancy? | 942 |
| Q221 At any time during the pregnancy, did asha ever come to your home to talk to you/ family members about you or your baby’s health? | 919 |
| Q243C In case of institutional delivery, did asha accompany you to the hospital? | 782 |
| Q301A1A Who provided you registration of pregnancy services?_ASHA | 768 |
| Q266 Did asha come to your home from hospital to drop you? | 631 |
| Q223 Besides these home visits, did you ever meet/had interaction with asha during your pregnancy? (health related interaction / outside home health related) | 542 |
| Q225 Did it ever happen that asha provided some health related advice for you to your relative (husband/mother in law etc)? OUTSIDE HOME | 457 |
| Q301E1A Who provided you services related to family planning-counselling home visits?_ASHA | 408 |
| Q242B12 If your last delivery happened in institution what were the reasons_ASHA’S SUPPORT | 240 |
| Q301F1A Who provided you filaria related services?_ASHA | 186 |
| Q301B1A Who provided you immunization services?_ASHA | 175 |
| Q301C1A Who provided you services related to newborn birh certificate?_ASHA | 148 |
| Q260I Did you receive any help from asha in getting this money? | 82 |
| Q205B11 If received 4 or more ANC’s, then why?_ASHA ENSURED/ACCOMPANIED | 55 |
| Q301G1A Who provided you malaria related services?_ASHA | 51 |
| Q207B9 If consumed 90 ifa tablets/3 syrup, why?_ASHA ENSURED THE AVAILABILITY | 39 |
| Q301D1A Who provided you services related to lbw identification/sam management?_ASHA | 39 |
| Q301H1A Who provided you services related to take home ration?_ASHA | 31 |
