# Supplementary material for: Facilitating behavioral change: A comparative assessment of ASHA efficacy in rural Bihar
Source: PLOS Glob Public Health. 2022 Aug 17;2(8):e0000756. doi: 10.1371/journal.pgph.0000756 (PMC10021476; doi:10.1371/journal.pgph.0000756)
Supplement: S2 Table — (DOCX) [file pgph.0000756.s004.docx]

Table S2: Logistic regression for overall effect of each influencer on the uptake of the 11 focal behaviors.

|  | OR | 2.5 % | 97.5 % | Estimate | Std. Error | z value | Pr(>\|z\|) |
| --- | --- | --- | --- | --- | --- | --- | --- |
| (Intercept) | 0.49 | 0.42 | 0.58 | -0.71 | 0.08 | -8.47 | 0.00 |
| ageclass20-24 | 0.91 | 0.81 | 1.04 | -0.09 | 0.06 | -1.38 | 0.17 |
| ageclass25-29 | 0.90 | 0.76 | 1.06 | -0.11 | 0.08 | -1.32 | 0.19 |
| ageclass30-34 | 0.89 | 0.72 | 1.10 | -0.11 | 0.11 | -1.05 | 0.29 |
| ageclass35+ | 1.07 | 0.81 | 1.42 | 0.07 | 0.14 | 0.50 | 0.62 |
| ageclass_married15-17 | 1.06 | 0.94 | 1.19 | 0.05 | 0.06 | 0.88 | 0.38 |
| ageclass_married18-20 | 1.10 | 0.97 | 1.26 | 0.10 | 0.07 | 1.46 | 0.14 |
| ageclass_married21+ | 1.13 | 0.89 | 1.43 | 0.12 | 0.12 | 0.97 | 0.33 |
| nkidscat2 | 0.97 | 0.86 | 1.09 | -0.03 | 0.06 | -0.52 | 0.60 |
| nkidscat3 | 0.89 | 0.77 | 1.02 | -0.12 | 0.07 | -1.65 | 0.10 |
| nkidscat4 | 0.87 | 0.73 | 1.04 | -0.14 | 0.09 | -1.54 | 0.12 |
| nkidscat5+ | 0.83 | 0.67 | 1.01 | -0.19 | 0.10 | -1.82 | 0.07 |
| educat1to7 | 0.89 | 0.79 | 1.01 | -0.11 | 0.06 | -1.77 | 0.08 |
| educat8to10 | 1.00 | 0.90 | 1.12 | 0.00 | 0.06 | 0.04 | 0.97 |
| educat11to13 | 1.05 | 0.88 | 1.24 | 0.04 | 0.09 | 0.51 | 0.61 |
| educat14to17 | 1.29 | 1.04 | 1.58 | 0.25 | 0.11 | 2.36 | 0.02 |
| wealthq2 | 1.04 | 0.92 | 1.18 | 0.04 | 0.06 | 0.64 | 0.52 |
| wealthq3 | 0.86 | 0.76 | 0.98 | -0.15 | 0.06 | -2.30 | 0.02 |
| wealthq4 | 0.92 | 0.80 | 1.04 | -0.09 | 0.07 | -1.31 | 0.19 |
| wealthq5 | 0.95 | 0.83 | 1.10 | -0.05 | 0.07 | -0.66 | 0.51 |
| Ifam | 1.98 | 1.82 | 2.15 | 0.68 | 0.04 | 16.27 | 0.00 |
| Ianm | 4.74 | 4.12 | 5.45 | 1.56 | 0.07 | 21.77 | 0.00 |
| Iasha | 6.16 | 5.56 | 6.84 | 1.82 | 0.05 | 34.43 | 0.00 |
| Idai | 1.10 | 0.92 | 1.31 | 0.09 | 0.09 | 1.04 | 0.30 |
| Ifriendrelnei | 1.20 | 1.09 | 1.33 | 0.19 | 0.05 | 3.70 | 0.00 |
| Iprivclinic | 2.26 | 1.91 | 2.68 | 0.82 | 0.09 | 9.40 | 0.00 |
| Igovdoc | 4.83 | 3.71 | 6.38 | 1.58 | 0.14 | 11.42 | 0.00 |
| Irmp | 0.39 | 0.25 | 0.59 | -0.94 | 0.22 | -4.32 | 0.00 |
| Imedia | 2.00 | 1.11 | 3.82 | 0.69 | 0.31 | 2.20 | 0.03 |
| IOTHER | 2.02 | 1.37 | 3.00 | 0.70 | 0.20 | 3.53 | 0.00 |
