# Supplementary material for: Facilitating behavioral change: A comparative assessment of ASHA efficacy in rural Bihar
Source: PLOS Glob Public Health. 2022 Aug 17;2(8):e0000756. doi: 10.1371/journal.pgph.0000756 (PMC10021476; doi:10.1371/journal.pgph.0000756)
Supplement: S3 Table — (DOCX) [file pgph.0000756.s005.docx]

Table S3: Results of selected logistic regression model that includes controls, influencers, behaviors, and influencer x behavior interactions

|  | OR | 2.5 % | 97.5 % | Estimate | Std. Error | z value | Pr(>\|z\|) |
| --- | --- | --- | --- | --- | --- | --- | --- |
| (Intercept) | 0.432 | 0.352 | 0.529 | -0.840 | 0.104 | -8.094 | 0.000 |
| ageclass20-24 | 0.924 | 0.806 | 1.059 | -0.079 | 0.070 | -1.135 | 0.256 |
| ageclass25-29 | 0.940 | 0.788 | 1.121 | -0.062 | 0.090 | -0.685 | 0.494 |
| ageclass30-34 | 0.882 | 0.704 | 1.106 | -0.125 | 0.115 | -1.087 | 0.277 |
| ageclass35+ | 1.124 | 0.829 | 1.524 | 0.117 | 0.155 | 0.751 | 0.453 |
| ageclass_married15-17 | 1.021 | 0.897 | 1.162 | 0.021 | 0.066 | 0.317 | 0.751 |
| ageclass_married18-20 | 1.108 | 0.961 | 1.278 | 0.103 | 0.073 | 1.411 | 0.158 |
| ageclass_married21+ | 1.170 | 0.902 | 1.518 | 0.157 | 0.133 | 1.183 | 0.237 |
| nkidscat2 | 0.920 | 0.809 | 1.045 | -0.084 | 0.065 | -1.282 | 0.200 |
| nkidscat3 | 0.861 | 0.742 | 1.000 | -0.149 | 0.076 | -1.960 | 0.050 |
| nkidscat4 | 0.846 | 0.703 | 1.019 | -0.167 | 0.095 | -1.764 | 0.078 |
| nkidscat5+ | 0.799 | 0.641 | 0.997 | -0.224 | 0.113 | -1.989 | 0.047 |
| educat1to7 | 0.936 | 0.816 | 1.073 | -0.066 | 0.070 | -0.946 | 0.344 |
| educat8to10 | 1.060 | 0.942 | 1.192 | 0.058 | 0.060 | 0.969 | 0.332 |
| educat11to13 | 1.210 | 1.006 | 1.455 | 0.190 | 0.094 | 2.025 | 0.043 |
| educat14to17 | 1.551 | 1.238 | 1.946 | 0.439 | 0.115 | 3.804 | 0.000 |
| wealthq2 | 1.113 | 0.975 | 1.271 | 0.107 | 0.068 | 1.589 | 0.112 |
| wealthq3 | 0.927 | 0.810 | 1.060 | -0.076 | 0.069 | -1.107 | 0.268 |
| wealthq4 | 1.017 | 0.883 | 1.171 | 0.017 | 0.072 | 0.234 | 0.815 |
| wealthq5 | 1.073 | 0.919 | 1.252 | 0.070 | 0.079 | 0.892 | 0.372 |
| Qancreg | 0.522 | 0.415 | 0.655 | -0.650 | 0.117 | -5.566 | 0.000 |
| Qcerealav | 4.176 | 3.105 | 5.641 | 1.429 | 0.152 | 9.392 | 0.000 |
| Qcolost | 4.094 | 3.071 | 5.479 | 1.409 | 0.148 | 9.552 | 0.000 |
| Qcordst | 2.081 | 1.574 | 2.751 | 0.733 | 0.142 | 5.152 | 0.000 |
| Qfast | 2.111 | 1.707 | 2.611 | 0.747 | 0.108 | 6.892 | 0.000 |
| Qhidep | 0.346 | 0.263 | 0.451 | -1.062 | 0.138 | -7.699 | 0.000 |
| Qifa | 0.122 | 0.088 | 0.166 | -2.102 | 0.162 | -12.967 | 0.000 |
| Qtibf | 0.503 | 0.375 | 0.667 | -0.688 | 0.146 | -4.695 | 0.000 |
| Qwork | 1.421 | 1.171 | 1.724 | 0.351 | 0.099 | 3.565 | 0.000 |
| Ifam | 3.072 | 2.707 | 3.488 | 1.122 | 0.065 | 17.359 | 0.000 |
| Ianm | 8.262 | 5.940 | 11.783 | 2.112 | 0.174 | 12.115 | 0.000 |
| Iasha | 10.146 | 8.211 | 12.628 | 2.317 | 0.110 | 21.112 | 0.000 |
| Idai | 0.870 | 0.649 | 1.166 | -0.139 | 0.149 | -0.932 | 0.352 |
| Ifriendrelnei | 1.821 | 1.516 | 2.192 | 0.600 | 0.094 | 6.382 | 0.000 |
| Iprivclinic | 2.174 | 1.606 | 2.968 | 0.777 | 0.156 | 4.964 | 0.000 |
| Igovdoc | 12.885 | 8.078 | 21.421 | 2.556 | 0.248 | 10.306 | 0.000 |
| Irmp | 1.740 | 0.907 | 3.436 | 0.554 | 0.339 | 1.637 | 0.102 |
| Imedia | 3.655 | 1.711 | 8.360 | 1.296 | 0.403 | 3.216 | 0.001 |
| Itraining | 4.982 | 1.580 | 19.169 | 1.606 | 0.623 | 2.576 | 0.010 |
| IOTHER | 1.685 | 1.022 | 2.811 | 0.522 | 0.257 | 2.028 | 0.043 |
| Qfast:Ifam | 0.261 | 0.198 | 0.344 | -1.343 | 0.141 | -9.513 | 0.000 |
| Qcordst:Ifam | 0.097 | 0.069 | 0.136 | -2.336 | 0.174 | -13.405 | 0.000 |
| Qhidep:Ifam | 0.260 | 0.178 | 0.377 | -1.347 | 0.191 | -7.053 | 0.000 |
| Qtibf:Ifam | 1.043 | 0.705 | 1.554 | 0.043 | 0.201 | 0.211 | 0.833 |
| Qcolost:Ifam | 0.277 | 0.194 | 0.396 | -1.284 | 0.182 | -7.071 | 0.000 |
| Qcerealav:Ifam | 0.384 | 0.280 | 0.527 | -0.956 | 0.162 | -5.907 | 0.000 |
| Qwork:Ianm | 0.169 | 0.071 | 0.431 | -1.775 | 0.457 | -3.886 | 0.000 |
| Qcordst:Ianm | 0.470 | 0.286 | 0.766 | -0.756 | 0.251 | -3.010 | 0.003 |
| Qhidep:Ianm | 0.054 | 0.018 | 0.136 | -2.925 | 0.513 | -5.701 | 0.000 |
| Qifa:Ianm | 1.305 | 0.750 | 2.267 | 0.266 | 0.282 | 0.945 | 0.345 |
| Qtibf:Ianm | 3.045 | 1.679 | 5.635 | 1.114 | 0.308 | 3.616 | 0.000 |
| Qancreg:Ianm | 0.268 | 0.154 | 0.462 | -1.318 | 0.279 | -4.719 | 0.000 |
| Qcerealav:Ianm | 0.198 | 0.102 | 0.395 | -1.618 | 0.343 | -4.713 | 0.000 |
| Qfast:Iasha | 0.827 | 0.326 | 2.550 | -0.190 | 0.515 | -0.369 | 0.713 |
| Qwork:Iasha | 0.467 | 0.263 | 0.867 | -0.761 | 0.304 | -2.505 | 0.012 |
| Qcordst:Iasha | 0.256 | 0.174 | 0.375 | -1.364 | 0.196 | -6.966 | 0.000 |
| Qhidep:Iasha | 0.202 | 0.125 | 0.321 | -1.601 | 0.241 | -6.635 | 0.000 |
| Qifa:Iasha | 1.699 | 1.095 | 2.661 | 0.530 | 0.226 | 2.340 | 0.019 |
| Qtibf:Iasha | 2.491 | 1.468 | 4.392 | 0.912 | 0.279 | 3.275 | 0.001 |
| Qcolost:Iasha | 0.769 | 0.455 | 1.358 | -0.263 | 0.278 | -0.947 | 0.344 |
| Qancreg:Iasha | 0.940 | 0.662 | 1.336 | -0.062 | 0.179 | -0.348 | 0.728 |
| Qcerealav:Iasha | 0.378 | 0.219 | 0.685 | -0.972 | 0.290 | -3.356 | 0.001 |
| Qcordst:Idai | 0.353 | 0.181 | 0.663 | -1.040 | 0.330 | -3.154 | 0.002 |
| Qtibf:Idai | 7.013 | 3.518 | 14.402 | 1.948 | 0.358 | 5.435 | 0.000 |
| Qcolost:Idai | 1.591 | 0.846 | 3.079 | 0.464 | 0.329 | 1.414 | 0.158 |
| Qcerealav:Idai | 0.258 | 0.150 | 0.438 | -1.355 | 0.273 | -4.968 | 0.000 |
| Qfast:Ifriendrelnei | 0.220 | 0.155 | 0.310 | -1.515 | 0.177 | -8.577 | 0.000 |
| Qwork:Ifriendrelnei | 0.768 | 0.534 | 1.108 | -0.264 | 0.186 | -1.418 | 0.156 |
| Qcordst:Ifriendrelnei | 0.191 | 0.114 | 0.312 | -1.655 | 0.256 | -6.462 | 0.000 |
| Qhidep:Ifriendrelnei | 0.569 | 0.349 | 0.907 | -0.563 | 0.243 | -2.320 | 0.020 |
| Qcolost:Ifriendrelnei | 0.609 | 0.391 | 0.959 | -0.495 | 0.228 | -2.170 | 0.030 |
| Qancreg:Ifriendrelnei | 1.283 | 0.851 | 1.947 | 0.249 | 0.211 | 1.180 | 0.238 |
| Qcerealav:Ifriendrelnei | 0.706 | 0.498 | 1.004 | -0.348 | 0.178 | -1.949 | 0.051 |
| Qwork:Iprivclinic | 12.814 | 4.993 | 43.620 | 2.551 | 0.539 | 4.732 | 0.000 |
| Qcordst:Iprivclinic | 0.136 | 0.076 | 0.238 | -1.994 | 0.290 | -6.868 | 0.000 |
| Qifa:Iprivclinic | 11.395 | 6.202 | 21.262 | 2.433 | 0.314 | 7.752 | 0.000 |
| Qtibf:Iprivclinic | 0.431 | 0.198 | 0.916 | -0.841 | 0.391 | -2.154 | 0.031 |
| Qcolost:Iprivclinic | 0.676 | 0.322 | 1.514 | -0.391 | 0.392 | -0.998 | 0.318 |
| Qancreg:Iprivclinic | 0.804 | 0.154 | 4.012 | -0.218 | 0.809 | -0.269 | 0.788 |
| Qcordst:Igovdoc | 0.138 | 0.069 | 0.272 | -1.980 | 0.350 | -5.664 | 0.000 |
| Qcolost:Igovdoc | 0.294 | 0.108 | 0.949 | -1.223 | 0.545 | -2.245 | 0.025 |
| Qcerealav:Igovdoc | 0.103 | 0.043 | 0.257 | -2.274 | 0.451 | -5.040 | 0.000 |
| Qcordst:Irmp | 0.042 | 0.011 | 0.133 | -3.162 | 0.627 | -5.040 | 0.000 |
| Qifa:Imedia | 0.188 | 0.036 | 1.240 | -1.670 | 0.891 | -1.875 | 0.061 |
| Qtibf:IOTHER | 1.420 | 0.384 | 5.173 | 0.350 | 0.658 | 0.533 | 0.594 |
| Qcolost:IOTHER | 1.818 | 0.439 | 12.485 | 0.598 | 0.815 | 0.733 | 0.463 |
| Qancreg:IOTHER | 1.221 | 0.307 | 5.069 | 0.200 | 0.705 | 0.284 | 0.777 |
