# Supplementary material for: Facilitating behavioral change: A comparative assessment of ASHA efficacy in rural Bihar
Source: PLOS Glob Public Health. 2022 Aug 17;2(8):e0000756. doi: 10.1371/journal.pgph.0000756 (PMC10021476; doi:10.1371/journal.pgph.0000756)
Supplement: S4 Table — (DOCX) [file pgph.0000756.s006.docx]

Table S4: Results of moderation analysis that includes controls and an interaction between each moderator and ASHA interaction score.

|  | Wealth | Caste | Religion |
| --- | --- | --- | --- |
| :-------------------------- | :----------------: | :----------------: | :----------------: |
| (Intercept) | 1.884*** | 1.877*** | 1.923*** |
|  | [1.836, 1.933] | [1.826, 1.929] | [1.856, 1.990] |
| Parity2 | -0.064* | -0.063* | -0.067** |
|  | [-0.114, -0.015] | [-0.112, -0.014] | [-0.116, -0.018] |
| Parity3 | -0.107*** | -0.111*** | -0.114*** |
|  | [-0.165, -0.049] | [-0.168, -0.053] | [-0.171, -0.056] |
| Parity4 | -0.097** | -0.103** | -0.106** |
|  | [-0.169, -0.024] | [-0.175, -0.031] | [-0.178, -0.034] |
| Parity5+ | -0.222*** | -0.230*** | -0.232*** |
|  | [-0.313, -0.131] | [-0.321, -0.140] | [-0.323, -0.142] |
| EDU1to7 | 0.030 | 0.031 | 0.037 |
|  | [-0.025, 0.085] | [-0.024, 0.086] | [-0.018, 0.092] |
| EDU8to10 | 0.081*** | 0.090*** | 0.093*** |
|  | [0.036, 0.126] | [0.046, 0.134] | [0.049, 0.137] |
| EDU11to13 | 0.149*** | 0.160*** | 0.168*** |
|  | [0.083, 0.215] | [0.097, 0.224] | [0.104, 0.231] |
| EDU14to17 | 0.218*** | 0.227*** | 0.236*** |
|  | [0.146, 0.290] | [0.158, 0.296] | [0.166, 0.305] |
| Age20-24 | 0.010 | 0.013 | 0.015 |
|  | [-0.043, 0.062] | [-0.039, 0.066] | [-0.038, 0.068] |
| Age25-29 | 0.043 | 0.053 | 0.052 |
|  | [-0.025, 0.111] | [-0.015, 0.120] | [-0.015, 0.120] |
| Age30-34 | 0.009 | 0.018 | 0.020 |
|  | [-0.082, 0.099] | [-0.073, 0.109] | [-0.071, 0.111] |
| Age35+ | 0.057 | 0.068 | 0.065 |
|  | [-0.066, 0.180] | [-0.054, 0.191] | [-0.058, 0.188] |
| ASHA_Int_c | 0.020*** | 0.025*** | 0.013** |
|  | [0.017, 0.023] | [0.020, 0.030] | [0.005, 0.022] |
| wealth_diff_c | -0.013+ |  |  |
|  | [-0.027, 0.002] |  |  |
| ASHA_Int_c × wealth_diff_c | 0.001 |  |  |
|  | [-0.001, 0.004] |  |  |
| caste_diff1 |  | 0.003 |  |
|  |  | [-0.032, 0.037] |  |
| ASHA_Int_c × caste_diff1 |  | -0.008* |  |
|  |  | [-0.014, -0.002] |  |
| relig_diff1 |  |  | -0.053+ |
|  |  |  | [-0.107, 0.001] |
| ASHA_Int_c × relig_diff1 |  |  | 0.008+ |
|  |  |  | [-0.001, 0.016] |
| Num.Obs. | 1186 | 1186 | 1186 |
| AIC | 4959.9 | 4958.1 | 4958.6 |
| BIC | 5046.2 | 5044.5 | 5045.0 |
| Log.Lik. | -2462.939 | -2462.072 | -2462.316 |
|  |  |  |  |
| __Note:__ |  |  |  |
| ^^ + p < 0.1, * p < 0.05, ** p < 0.01, *** p < 0.001 |  |  |  |
