# Supplementary material for: Facilitating behavioral change: A comparative assessment of ASHA efficacy in rural Bihar
Source: PLOS Glob Public Health. 2022 Aug 17;2(8):e0000756. doi: 10.1371/journal.pgph.0000756 (PMC10021476; doi:10.1371/journal.pgph.0000756)
Supplement: S5 Table — (DOCX) [file pgph.0000756.s007.docx]

Table S5: Results of moderation analysis that includes controls and an interaction between each moderator and ASHA interaction score.

|  | No home visit | Ho home visit and no outside visit |
| --- | --- | --- |
| :------------- | :----------------: | :----------------------------------: |
| (Intercept) | -1.156*** | -1.723*** |
|  | [-1.747, -0.591] | [-2.453, -1.045] |
| Parity2 | 0.194 | 0.204 |
|  | [-0.221, 0.612] | [-0.297, 0.709] |
| Parity3 | 0.080 | -0.021 |
|  | [-0.403, 0.564] | [-0.619, 0.574] |
| Parity4 | -0.102 | -0.278 |
|  | [-0.712, 0.500] | [-1.059, 0.478] |
| Parity5+ | 0.062 | 0.175 |
|  | [-0.667, 0.772] | [-0.704, 1.024] |
| EDU1to7 | -0.421+ | -0.515 |
|  | [-0.924, 0.048] | [-1.179, 0.076] |
| EDU8to10 | 0.120 | 0.038 |
|  | [-0.245, 0.482] | [-0.411, 0.477] |
| EDU11to13 | -0.146 | -0.222 |
|  | [-0.763, 0.432] | [-0.992, 0.475] |
| EDU14to17 | -0.467 | -0.350 |
|  | [-1.253, 0.229] | [-1.293, 0.459] |
| Age20-24 | -0.141 | -0.197 |
|  | [-0.574, 0.299] | [-0.717, 0.335] |
| Age25-29 | 0.069 | 0.012 |
|  | [-0.476, 0.618] | [-0.648, 0.678] |
| Age30-34 | -0.238 | -0.463 |
|  | [-0.993, 0.493] | [-1.448, 0.456] |
| Age35+ | -0.488 | -0.158 |
|  | [-1.619, 0.520] | [-1.428, 0.967] |
| wealth_diff_c | 0.107+ | 0.034 |
|  | [-0.013, 0.228] | [-0.113, 0.181] |
| caste_diff1 | -0.290* | -0.177 |
|  | [-0.573, -0.005] | [-0.525, 0.173] |
| relig_diff1 | 0.106 | 0.089 |
|  | [-0.335, 0.576] | [-0.443, 0.681] |
| Num.Obs. | 1186 | 1186 |
| AIC | 1254.3 | 933.7 |
| BIC | 1335.5 | 1015.0 |
| Log.Lik. | -611.134 | -450.863 |
| F | 1.280 | 0.650 |
|  |  |  |
| __Note:__ |  |  |
| ^^ + p < 0.1, * p < 0.05, ** p < 0.01, *** p < 0.001 |  |  |
