# Supplementary material for: Facilitating behavioral change: A comparative assessment of ASHA efficacy in rural Bihar
Source: PLOS Glob Public Health. 2022 Aug 17;2(8):e0000756. doi: 10.1371/journal.pgph.0000756 (PMC10021476; doi:10.1371/journal.pgph.0000756)
Supplement: Attachment — Submitted filename: PLOSGPH revision1 response to reviewers.docx [file pgph.0000756.s008.docx]

Dear Editors of PLOS Global Public Health,

Thank you for considering our manuscript, “Facilitating behavioral change: A comparative assessment of ASHA efficacy in rural Bihar" for publication in your journal.

We found the reviewer comments very helpful and addressing them has greatly improved the quality and clarity of the paper. Thank you for facilitating such helpful reviews.

First, we make an overall summary of revisions and then we respond to each concern by both reviewers in kind.

To revise this paper, we took great effort to shorten and clarify. We moved figures and results to the SOM to simplify the main text and we drew sharper distinctions between results and methods. Previously, we included some references to the results in the Methods section in order to clarify the nature of the coding, but removing this material does simply the presentation. Much of the moving and cutting of material was to address Reviewer 1’s overall concerns about manuscript length and being sure to not refer to results in the methods section.

The analysis under question 3, about ASHA-Mother differences, was mostly moved to the SOM, in the interest of space. We also moved the figures that showed the percentages of association of each influencer with each behavior to the SOM. There was quite a bit of text associated with these figures so making this move also helped shorten the manuscript.

Reviewer 2 had a concern about causal language in the paper. We very much agree with this concern and have taken care to make sure that overtly causal language is not present. One term that may have been unclear is the use of the term ‘effect’. For us, if we are referring to the result of a regression model one can say X has an effect on Y and simply mean the estimate of the coefficient of X on Y without asserting a causal effect. In most cases we use either “correlation” or “association” to describe the statistical relationship between a given influencer and a given behavior. We attempted to remove all overtly causal language, in agreement with the reviewer on this point.

The detailed responses follow:

*****************************************************************************************

REVIEWER 1: Comments and Responses

*****************************************************************************************

Reviewer #1: Dear Authors,

Thank you for taking the time to do this elaborate study - on an important topic. I commend you on taking up a very elegant study design.

- Thank you very much for commending the study design.

My concerns are primarily with the organization of the paper and the readability of it.

I encourage you to organize the material better-

a) The introduction is too elaborate- can you cut it down and emphasize why specifically you did this study?

- Yes, we agree that the Intro was too elaborate. To shorten the introduction, we have:
  - Reduced detail on the ASHA program and on the background of research on the topic. We also moved the paragraph about influencers to the methods section where we explain how influencers were coded, which is a much better location for this material.

b) With the elegant study design - I got lost in the language and the places where the methods and results merged. I would suggest a section called "Instrumentation" - that crisply describes the process and the specific survey tools developed.

- Please refrain from adding results to this.

- We have ensured that methods and results are indeed in different sections. For instance, we removed the counts and percentages from behaviors column of Table 1 and removed references to counts or percentages in the explanation of how behaviors were coded. We initially renamed the methods section to “Instrumentation”, but we hope the reviewer will understand that the heading “Materials and Methods” is specifically called for in the journal’s formatting guidelines and as a result we unfortunately had to turn it back. We apologize for not being able to make that exact change. However, we were sure to keep the information under this header much more strongly focused on methods. The Results section has a separate header.

c) The results section is confusing partly because of too many tables/figures. Could you simplify these? It would enhance the readability significantly.

- We have moved three tables/figures from the results to the supplementary materials (SOM). We also attempted to streamline the presentation of the results and removed some of the commentary. Our hope was that by keeping the results organized by research question that they wouldn’t be too confusing. We hope it is better now.

d) Please do not bring in discussion elements into the results section.

- We have revised the results section and attempted to remove discussion elements from it.

e) The discussion section has a lot of richness. I wish it were more succinct. I felt like I was reading an elaborate results section rather than a discussion section.

- Thank you – we do indeed have fairly elaborate results. We have done our best to shorten the discussion section.

Again, there is a lot of richness in this manuscript. This is a field I am very familiar with. However, it is a very difficult one to read. I would strongly recommend simplifying the writing to reach your readers.

- Thank you – we hope that the manuscript is improved after these revisions. Thank you for your helpful comments.

*****************************************************************************************

REVIEWER 2: Comments and Responses

*****************************************************************************************

Reviewer #2: Hi, Please find attached the manuscript with my comments as ‘sticky notes’ in the attached document.

My over all comment: publishable only after these comments are addressed. Also, need to simplify the way the graphs are explained in results. And not attribute causation.

- Thank you. We have done our best to address these concerns.
- Note: the following locations of the sticky notes apply to the original submitted pdf – the line numbers don’t match up to the tracked changes version because this shifted the text around. We supply the exact text of each sticky note to ensure clarity with how each comment was addressed.

Sticky note comments:

1) Page 2, Abstract, lines 26-17 – This maybe misleading and is factually incorrect

- The sentences near this sticky note are: “ASHAs specialize in maternal and child health but also support other health initiatives. Like other CHWs, ASHAs are associated with many improvements in health behaviors.”
  - While neither statement is factually incorrect we assume that statement taken as misleading is “ASHAs are associated with many improvements,” but we cannot be certain.
  - But just to be certain, we have completely rephrased both of these sentences to highlight the fact that many studies of ASHA efficacy have been conducted and the results suggest mixed results because for us the important point is the difficult in drawing prevailing impressions from existing evaluations. We hope that the revisions have helped clarify this point.

2) Page 2, Abstract, lines 37 - There is a mention that this field suffers from a narrow foucs on select behaviors only and yet the authors refer to only the infuence on perinatal behavors here.

- Thank you for allowing us to clarify. We include more totally behaviors than any study that we are aware of and certainly more than are typical of ASHA evaluations. Maternal and child health behaviors are often emphasized ASHA duties. Earlier in the abstract we point out that ASHAs also engage in other tasks and this point is returned to in the Discussion. We agree that it would be interesting to have more data on how non-maternal-and-child-health duties affect ASHA workload and efficacy.

3) Page 2, Abstract, line 40 – Unlclear

- We have adjusted phrasing and attempted to clarify this statement.

4) Page 9, line 180 – Good to clarify-random or non-random

- Yes, good point. We have added language to clarify how mothers were recruited into the study within each AWC. They were indeed randomly selected (with condition that a mother had given birth within previous six months).

5) Page 11, line 229 - non-bathing?

- Phrasing corrected to ensure emphasis that the recommendation is to not bathe the infant within 24 hours of birth.

6) Page 28, line 488 – 489 - Which response variable?

- The response variable is a binary variable coded as 1 if the mother adopted the biomedically recommended behavior and 0 if she did not.

7) Page 30, Figure 3 - Combining the background variables and 'exposure' variables in OR slide confuses the reader on central point of this figure-who is the most effective infuencer.

- We have redrafted the figure to show only the influencer categories and exclude the control variables.

8) Page 31, line 517 - 518 - Language edit required

- We were not sure what this comment referred to but we did our best to change the language in the first two sentences of this paragraph.

9) Page 32, line 538 - Can we really say this? Do we have information/data to substantiate? Not sure. Maybe better to state this is a limitation of the survey q

- This seems to be in reference to this sentence: “For instance, if a woman registered her pregnancy in month 4, which is scored as not consistent with biomedical recommendations, she may not be considered to have missed a target and in turn, the association with an ASHA as a form of influence is not equivalent to the ASHA being a barrier to the recommended behavior.”
- The intent is to make it clear that a causal assertion cannot be made. However, in editing this sentence has been deleted from the manuscript.

10) Page 32, line 540 - while doctors have been mentioned sparingly as an influence here, it is interesting to see that they have the strongest influence as per the modelling estiates on figure. This may merit a mention in the description of fig 5.

- Thank you for pointing out that there are many implications of our analysis that go beyond what we could cover in the paper. Indeed, we hope that readers will find results like those estimates across behaviors and sources as points-of-reference for other interests or applications.
- The focus of this paper is the ASHA, but we agree that it is important to frame ASHA influence in reference to a range of other possible sources of reference.
- In the overall result, the government doctor does not have the strongest influence, by means of point estimate, but certainly has overlapping error bars with the ASHA. For the results that separate by behavior, the government doctor certainly does have the strongest influence for some of the behaviors and this is excellent to see. We are not aware of a debate surrounding doctor efficacy, however, as there is for ASHAs, which is what motivated our study. The paper is already fairly long and we are concerned what additional discussion of other influencer categories would do to focus and length. But we completely agree with the reviewer that this is an important result.

11) Page 34, line 564 - Does this mean ASHAs different ASHAs have different influence or is the finding a fn of the survey design?

- The finding is that ASHAs were sometimes the second most positive source of influence or sometimes the third. Being able to rank the ASHA’s influence for each behavior is a function of the survey design and the analysis plan. We do not directly compare different ASHAs, but this must be an important factor in a general sense.

12) Page 37, lines 604 – 605 - This seems to be author speculations

- This comment seems to be in reference to this sentence: “Across each of the behaviors considered here, the ASHA is always nudging behavior in biomedically-desirable directions, and her effect is always more positive than sources of normative influence like family or friends/relatives/neighbors.”
- We cannot be certain which sentence the sticky note applied to but if this is indeed the statement then we would point out that it is based on the model-predicted probabilities displayed in Figures 5 and 6 (Figures 6 and 7 in the reviewed draft). For every behavior examined, the ASHA has a positive influence and for every behavior examined the ASHA’s influence is more strongly positive than family or friends/relatives/neighbors. Of course we do not know if this finding would be found in other datasets, but for this particular analysis it is a direct restatement of the results of the regression model. We are not sure what part of the statement seems speculative, but we adjusted the sentence to make it clear where the support for it comes from.

13) Page 46, lines 677 – 678 - This is a 'causal' reference, the best the data captured can infer is strong association.

- We very much agree with the general point but we are not quite certain what phrase or word prompted it. This comment comes just above a sentence that says: “Our results indicate strong positive correlations between the uptake of recommended behaviors by mothers and either having contact with ASHAs or being influenced by them.” To us, emphasizing the term ‘correlation’ here would seem to make it not a causal reference. We don’t see a major difference in meaning between the words ‘correlation’ and ‘association’, at least not in terms of avoiding a causal claim.
- We agree with the reviewer that there is indeed a strong statistical association between mothers who named ASHAs as a source of influence and the uptake of biomedically recommended behaviors.
- The statement in question is a restatement of empirical results. Causality in this setting would be hard to determine, or even define, and would require a different study design.
- Later on in the Discussion we also say “…begs the question of what is responsible for the association between ASHAs and the uptake of recommended behaviors.” – which contains the term ‘association’.
- And again in the Discussion we use the term ‘broad association’. As such, we are not sure where the causal reference occurs, but we agree that there is a strong statistical association present.
- In a few cases we use the term ‘effect’ meaning the statistical estimate. We have replaced some of these with the term ‘association.’

14) Page 47, line 697 - the discussion need not include reference to the results

- We are not sure if we understand this comment and are not sure of the action to take. The discussion is where one discusses the results of the paper and from our experience it is extremely common to refer back to figures and results in discussion sections. We agree that it perhaps could be overdone (one could overstate results in a discussion), but it is generally helpful to make some direct linkages so that the reader knows which result is being referred to in the discussion.
- That said, in the course of editing some of the figure references were cut.

15) Page 48, line 705 - ref?

- This seems to be a request for a reference. This sentence is the topic sentence for the first paragraph of a discussion section. The support for the statement is in the present paper. The support for it is stated in the sentences that follow it in the paragraph.

16) Page 48, line 705 - ? Bihari?

- Bihari means ‘a native or inhabitant of Bihar’. Bihar is where the study takes place.

17) Page 48, line 720 - ? see comment below

- Okay

18) Page 49, line 743 - While I appreciate the reference of ASHA as a program construct, the authors need to explain their thinking before making this reference. Technically, ASHA is an activist-referring her as a program may not be understood by the readers.

- We have added a good deal of text to elaborate on and clarify why we think the ASHA-as-person vs ASHA program distinction is important.
- We agree that by original rationale, the individual ASHA is intended to be an activist (whether or not that is aspirational or realized is a subject of discussion). Indeed, part of the broader point here is that the ability of individual ASHAs to reach their potential as activists, or behavior change agents, may sometimes be hampered by program-level constraints or that program issues sometimes get misattributed to the efficacy of ASHA workers. Evaluations of ASHA efficacy often conflate program and person in ways that make it difficult to draw general conclusions about what underlies shortcomings in service access or delivery. We think that this distinction is important to draw. We have added language in an attempt to clarify the point.
- The National Rural Health Mission (NRHM) of India established the ASHA program in 2005. The second A in ASHA does indeed stand for Activist. But this does not mean that there is no program. The ‘ASHA Program’ is encompassing effort that established the training, guidelines, and expectations for what ASHAs do, how they are incentivized, etc.
- Here is a helpful reference: <https://nhm.gov.in/images/pdf/communitisation/task-group-reports/guidelines-on-asha.pdf>
- Many papers raise the issue of whether or not they think ASHAs are living up to the ideal of being activists, due to a focus on service extension. There is more to this discussion than we can cover in the space of the paper but we do cite some of these papers and agree that the topic is both important and complex.

In closing, we again thank the editor and both Reviewers for their constructive and helpful commentary. We find that addressing the reviewer concerns has greatly improved the quality of the paper and appreciate the suggestions very much.

Best wishes,

Oskar Burger

**OSKAR BURGER, PH.D.**

Director of Design & Development | Center for Applied Cognitive Science

The University of Texas at Austin | Department of Psychology | centerforappliedcogsci.com
